# Improving conservation effectiveness of nature reserve for golden snub‐nosed monkey, a niche‐based approach

**DOI:** 10.1002/ece3.4447

**Published:** 2018-08-24

**Authors:** Zhaogui Yan, Mingjun Teng, Wei He, Yuan Wang, Jingyuan Yang, Pengcheng Wang

**Affiliations:** ^1^ College of Horticulture and Forestry Sciences/Hubei Engineering Technology Research Center for Forestry Information Huazhong Agricultural University Wuhan China; ^2^ Hubei Province Key Laboratory of Conservation Biology for Shennongjia Golden Monkey Shennongjia National Nature Reserve Moyu China

**Keywords:** environmental management, golden monkey, Maxent model, reserve selection, Shennongjia, wildlife conservation

## Abstract

Reserve selections are often opportunistic rather than strategic and coordinated, and consequently, many reserves are ineffective to achieve their intended goals of conservation. Here, we assessed the conservation effectiveness of a reserve for the golden snub‐nosed monkeys (*Rhinopithecus roxellana*) with a niche‐based approach. We assessed habitat usage of the monkeys in Shennongjia Nature Reserve (SNR) and attributes of 14 environmental variables that could potentially affect the monkeys’ habitat use. Spatial distribution of potentially suitable habitat for the monkeys was then modeled with Maxent, a niche‐based model, and conservation effectiveness of SNR was assessed by comparing the current boundary of the reserve with the spatial distribution of the modeled potential habitat and the current habitat area of the monkeys. Only 59% of the habitat area and 61% of the predicted potential habitat area were under the protection of SNR. To improve conservation effectiveness of SNR, we proposed that the current SNR be enlarged by 270 km^2^. The enlarged reserve would encompass 100% of the existing habitat area plus 89% of the predicted potential habitat area. Using the niche‐based approach, we were able to integrate habitat usage data of the target species with that of remote sensing to identify areas potentially suitable as habitat for the species. This information can be used not only for improving conservation effectiveness of existing reserves but also for the effective planning and designing of new reserves.

## 1 INTRODUCTION

Protection of natural habitat has been one of the most effective means of *in situ* conservation of endangered species and biodiversity (Chape, Harrison, Spalding, & Lysenko, 2005; Le Saout et al., 2013; Myers, Mittermeier, Mittermeier, Da Fonseca, & Kent, 2000). In recognition of the importance of biodiversity conservation, large areas worldwide have been set aside since 1950s as nature reserves or national parks. About 13% of the Earth's land area is under protection for the benefit of biodiversity conservation and ecological services (Kemsey et al., 2012). In China, protected areas of various categories cover about 15% of the total land area, that is, about 1.5 million km^2^ (Huyan, Xiao, Yu, & Xu, 2014). Activities that negatively impact on the health of the environment are generally prohibited within these protected areas. The effectiveness of these reserves to achieve their intended goals of conservation, however, is often compromised by two factors. First, it is not always possible to select areas of highest conservation values as reserves. In fact, most reserve selections are often opportunistic rather than strategic and coordinated (Pressey, Humphries, Margules, Vane‐Wright, & Williams, 1993; Pressey & Tully, 1994). In their study of the Australian reserve systems, Pressey and Tully (1994) found that most of the reserves are on land with the least potential for commercial use rather than on land with the highest conservation value. Ad hoc reserve selection is also considered to be the norm in the United States and other countries (Holdgate, 2014; Runte, 1997). The lack of accurate information on the habitat requirements of the target species and climate change further undermines the effectiveness of selected reserves (Araújo, Cabeza, Thuiller, Hannah, & Williams, 2004; Pavez‐Fox & Estay, 2016; Rondinini, Stuart, & Boitani, 2005; Sieck, Ibisch, Moloney, & Jeltsch, 2011). Pavez‐Fox and Estay (2016) analyzed the national reserve network of Chile and found that existing reserves are ineffective for the conservation of pudú, an endangered deer species endemic to South America, despite the fact that over 19% of the country is under protection.

Second, for conservation of endangered species, one of the primary goals was to build up as large a population(s) as possible to the point that they are no longer threatened with extinction (National Research Council, 1995). As population of endangered species changes in response to conservation management, their habitat requirements may change, rendering existing reserves ineffective for their conservation. Thus, there is a continuous need to review existing reserves in light of the changed population dynamics of the species under conservation.

Here, we assessed conservation effectiveness for the golden snub‐nosed monkey (*Rhinopithecus roxellana*), using Shennongjia Nature Reserve (SNR) as our model of study. The golden monkey is best known as China's second national treasure (second only to the giant panda: Wang, Jian, & Li, 1998; Li et al., 2007). The species is on the endangered species list of a number of organizations including the Endangered Wildlife Annex I of Convention on International Trade of Endangered Species (Favre, 1989), China Red Data Book of Endangered Animals (Sung, Peiqi, & Yiyu, 1998; Wang & Xie, 2004), and Vulnerable Species of International Union for Conservation of Nature and Natural Resources (IUCN 2013). Habitat conservation and management of the golden snub‐nosed monkey is one of the highest priorities in wildlife conservation in China (IUCN 2013). SNR was established in the 1980s for conservation of biodiversity of the area with the primary aim of providing a refuge for the golden snub‐nosed monkeys, as well as other animal and plant species (Wang, 1995). At the time of reserve establishment, relatively little was known about the population dynamics of the flagship species, the spatial distribution of its habitat areas, and how these will respond to conservation management. While the boundary of the reserve has remained unchanged since its first enactment more than 30 years ago, the population of the monkeys and vegetation have undergone marked changes in response to conservation management and climate change, further necessitating the need to examine the effectiveness and adequacy of the reserve.

In China, nature conservation is often synonymous with the cessation of all existing agricultural and forestry activities, whereas activities such as recreation and road traffic are often considered to have a minimum impact on nature conservation and are thus permitted in many of the nature reserves. While this assumption is largely correct for most plant and animal species, species like the golden snub‐nosed monkey, which has specific requirements on temperature, food and social space, reserve selection, and management needs to take these requirements into consideration. Since its enactment in 1983, SNR was protected from hunting, agriculture, and forestry activities. The population of the golden snub‐nosed monkey in the study region fluctuated, and by 2008, reached 1,200 (Xiang et al., 2011). Our preliminary field observations revealed that activities of the monkey were confined to particular section of the reserve, and the rest of the reserve was not utilized.

To study habitat usage of the monkeys, a team of field patrol followed the footprint of known group of monkeys during 2012–2013. We used the Maxent model for data analysis and modeling the spatial distribution of potential habitat for the golden snub‐nosed monkey for winter–spring (November–May) and summer–autumn (June–October). The Maxent model builds on the concept of maximum entropy of ecological niche (Phillips, Anderson, & Schapire, 2006). The model uses data of biotic and abiotic variables to calculate the Habitat Suitability Index (HSI) of given site or area for the target species. One advantage of this approach is its ability to use only the known geographic distribution data of a target species and distribution point corresponding to the environmental variables (i.e., “presence”) to inversely calculate the environmental requirements of the species (Phillips & Dudík, 2008). Using presence‐only data, the model is able to identify areas where no presence records currently exist but where the biotic and abiotic variables both satisfying the requirements of the target species. Combined with GIS technology, the model provides an effective means of evaluating spatial variability in habitat conditions of target species and produces habitat maps that can be used by policymakers and field managers of nature reserves.

To assess the conservation effectiveness of SNR for *R. roxellana*, we compared the current boundary of the reserve with the spatial distribution of the modeled potential habitat and the habitat area of the species. We attempted to answer the following two questions: (a) Are we preserving the areas with the greatest conservation value and (b) how can management practice be changed to improve conservation effectiveness of the existing reserve?

## 2 METHODS

### 2.1 Study area

**Figure 1:**
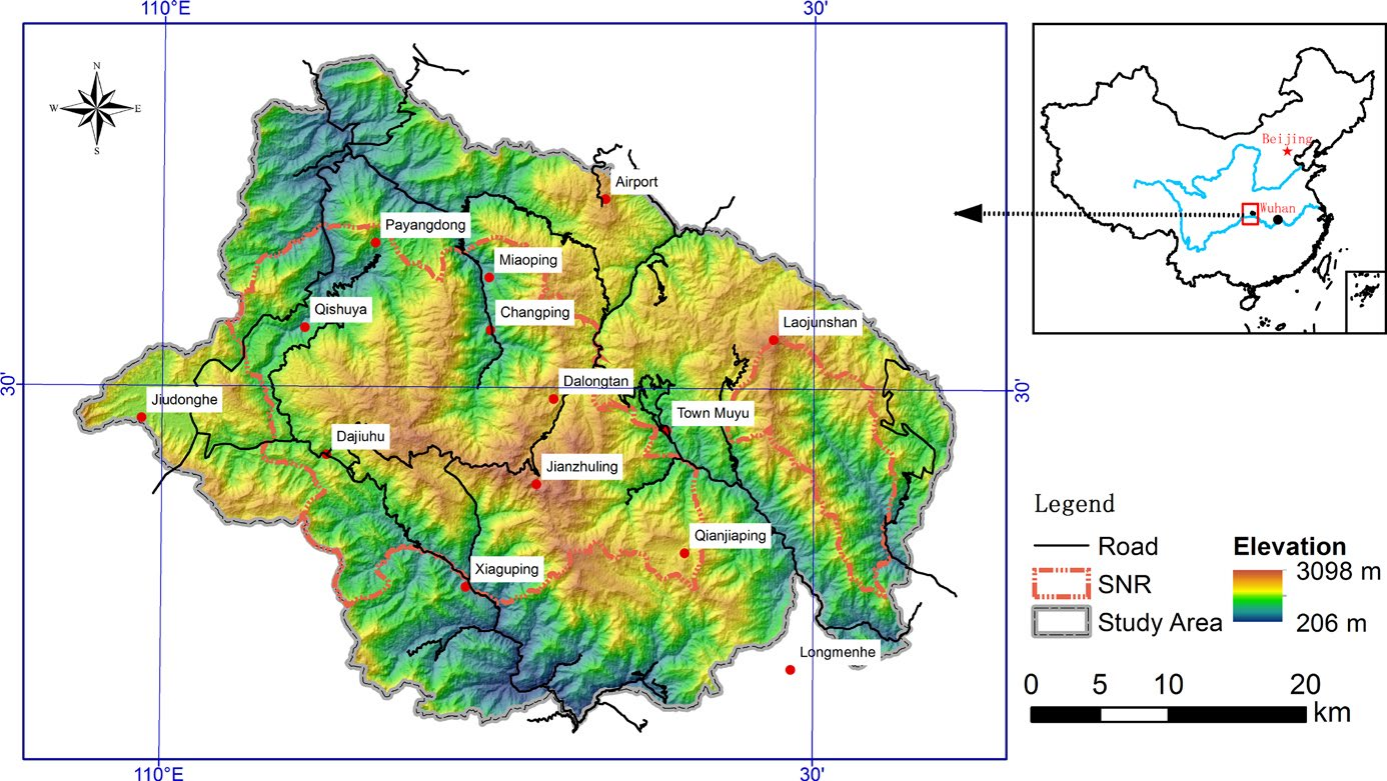
Topographic map of Shennongjia District, Hubei, China, showing the boundary of Shennongjia Nature Reserve (SNR). Elevation of the study area ranges from 200 to 3,100 m above sea level

Our study was conducted at Shennongjia District, Hubei, China (Figure 1). Total size of the study area was 1,797 km^2^, 722 km^2^ of which was in SNR (Zhu & Song, 1999). Shennongjia is located in the subtropical monsoonal region of China. Its climate is dominated by the monsoonal atmospheric circulation, and temperature is at the lower range of the subtropical region with a prolonged rainy season during summer–autumn period (May–October). Vegetation has formed local vertical zonation. Three climatic zones, low‐mountain, mid‐mountain, and subalpine, are recognized locally as elevation increases. Average annual temperature is 12°C with large daily temperature differences. The vegetation in the area consists mainly of subtropical elements, with minor elements of temperate and tropical origin. Average elevation of the region is 1,700 m above sea level (ASL), with many mountain peaks over 1,500 m ASL with six raising above 3,000 m ASL. The area is renowned for its richness in natural vegetation and wildlife resources.

### 2.2 Data collection and analysis

Field activity data of the golden snub‐nosed monkey were collected in SNR by the field petrol team during 2012–2013. When individuals of the monkeys were sighted, the data collected were as follows: counts of individuals, animal feces, and marks of scratches and bites. The geographic coordinates at each data collection point were recorded with a hand‐held GPS. Duplicate records from the same location were removed, leaving only one record for each grid cell (30 × 30 m). In all, 1,199 data sets were collated from the field patrols.

**Figure 2:**
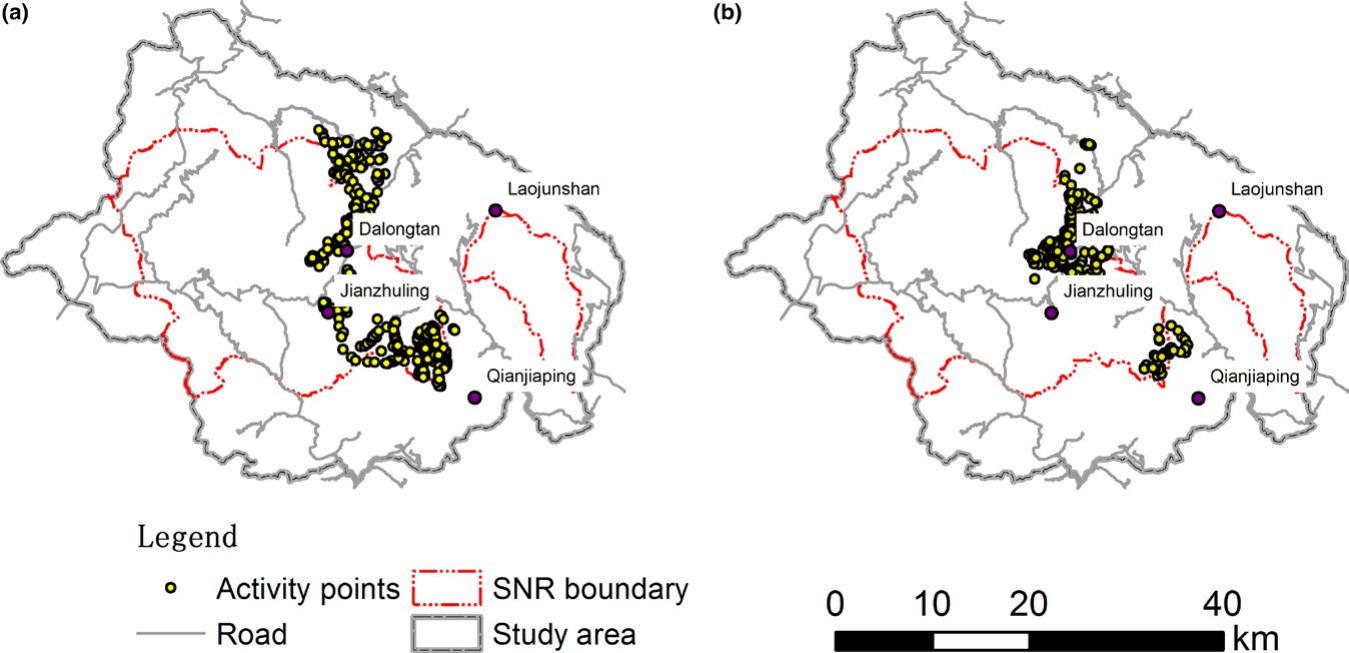
Spatial distribution of field activity points of *Rhinopithecus roxellana* in Shennongjia District, Hubei, China. SNR: Shennongjia Nature Reserve. Total activity points: 661 for summer–autumn (a) and 538 for winter–spring (b)

*Rhinopithecus* spp. are known to select different habitat and food between summer–autumn and winter–spring periods (Li, 2006; Li, Stanford, & Yang, 2002). Our preliminary analysis of the data indicated that activities of the golden snub‐nosed monkey differed both spatially and between winter–spring and summer–autumn (Figure 2). During summer–autumn, activity points were widely spread between areas north and south of Jianzhuling (Figure 2a) with 29% of the activity points outside SNR. In comparison, winter–spring activity points were congregated in two areas, the Dalongtan area in the north and a small area at the southeast corner of the reserve (Figure 2b) with 12% of the activity points outside SNR. Data of the two periods were therefore treated separately in all subsequent analyses.

All activity data were entered into ArcGIS10.0 for mapping and converting to formats that are compatible with the Maxent niche model. Data for 14 environmental variables, including three topographic, nine ecological, and two human‐related, were collated for the study area (Table 1). The digital elevation model (DEM) data used in this study were from the 30 × 30 m resolution data of the US Geological Survey website (http://www.usgs.gov). The DEM data were input into ArcGIS10.0. Elevation, aspect, slope, distance to the river, and other geographic data were then extracted using the spatial analysis capability of the software. Road data were provided by the SNR authority. The road vector data were converted to raster data in ArcGIS 10.0, and grid layer data were derived through linear distance analysis of the raster data.

**Table 1:** Description of the 14 environmental variables that affect habitat suitability for *Rhinopithecus roxellana* in Shennongjia Nature Reserve (SNR) and surrounding areas of Shennongjia District, Hubei, China

Type of data	Description of data	Source of data
*Golden monkey activity data*		
Activity points	Counts of individuals sighted, animal feces, marks of scratches, and bites	Field survey, routine field patrols
*Other variables*		
Topographic	Elevation, Slope, Slope aspect,	http://www.usgs.gov
Human disturbance related	Distance to water, Distance to road	SNR authority
Ecological	Tree layer canopy cover, Tree height, Tree DBH (diameter at breast height), Shrub height, Shrub cover, Herbal height, Herbal cover	National Forest Resource Database (8th edition)
	Land use/vegetation type	Landsat‐8 images

To quantify habitat characteristics of the monkeys, attributes of seven ecological variables (Table 1) were extracted from the National Forest Resource Database for Forest Management Planning and Design (8th Edition, 2009–2013). The data were converted into pixel data in ArcGIS 10.0. The NDVI data were extracted using IMAGINE ERDAS 9.2. Data on land use and vegetation types of the study area were derived from the Landsat 8 images and were grouped into six categories following the classification of Liu et al. (2014), viz. coniferous forest, mixed coniferous and broad‐leaved forest, shrub forest, coniferous forest, alpine meadow, and inhabited area. The demarcation of each land use and vegetation category was conducted in ERDAS IMAGE 9.2. Where possible, identification and distribution of vegetation types were verified with data from our field survey and from inventories of the SNR authority. The data were entered into ArcGIS10.0 for distance and area estimations for each of the land use/vegetation categories. Using ArcGIS 10.0 as the platform, the coordinates of each environmental variable were projected to the coordinate system, WGS 84/UTM zone 49N, and converted into ASC file format. The data were then used as input for the Maxent model for further analysis and modeling.

### 2.3 Maxent model

The Maxent model is widely used in wildlife management to define relations between species distribution and ecological variables (Chang et al., 2012; Morrison, Marcot, & Mannan, 2012; Radosavljevic & Anderson, 2014). While its application in the study of the golden snub‐nosed monkey has not been attempted before, our preliminary analysis suggested that results of the Maxent model were consistently better than models such as Bioclim or DOMAIN (Baldwin, 2009; Kumar & Stohlgren, 2009; Thorn, Nijman, Smith, & Nekaris, 2009). We used MAXENT 3.3.3k for our model development. The modeling software uses the receiver operating characteristic (ROC) curve and the subject area under the ROC curve (AUC) to verify the precision of the model forecast. One advantage of this approach is that it provides a single measure of model performance, independent of any particular choice of threshold. The higher the AUC values for given environmental variable, the closer the correlation between the variable and the geographic distribution model of the target species, and the better the forecast (Baldwin, 2009; Phillips et al., 2006). The output of the model is an index that reflects the suitability of habitat, that is, HSI ranging from 0 (areas least suitable as habitat) to 1 (areas most suitable as habitat) for given areas.

Various methods for classification of habitat suitability have been used and most of which are experience‐based rather than precise science (Liu, Newell, & White, 2016; Liu, White, & Newell, 2013). We recognized three habitat classes which were verified by direct field observations: areas not suitable as habitat (HSI = 0–0.2), low suitability habitat (HSI = 0.2–0.5), and high suitability habitat (HSI > 0.5). HSI maps were produced using ArcGIS 10.0. Separate maps were produced for winter–spring and summer–autumn and for the two periods combined. The area in each HSI class was calculated following the methodology of Kumar and Stohlgren (2009) and Han et al. (2014).

## 3 RESULTS

The effects of 14 variables on habitat suitability of *R. roxellana* were evaluated using Maxent model. During summer–autumn, the top six variables (Elevation, Tree cover, Shrub height, Slope, Distance to water, and Distance to road) accounted for up to 88% of the habitat suitability variability (HSV). During winter–spring, the top six variables accounted for up to 85% of the HSV; Elevation, Shrub height, Shrub canopy cover, Tree size height, Slope, and Distance to water made up the top six variables, whereas Distance to road (and water to a lesser degree) became less important during summer–autumn (Table 2). Elevation remained the most influential factor, accounting for 42% and 26% of the HSV for winter–spring and summer–autumn periods, respectively (Table 2). During winter–spring, the habitat areas were restricted to elevation range 2,200–2,800 m ASL. During summer–autumn, the lower limit of the elevation range reached 1,750 m ASL, whereas the upper limit reached 3,100 m ASL. Contrary to our expectation, the effects of vegetation type on HSV were small for both summer–autumn and winter–spring periods. One plausible explanation was that vegetation type was correlated with other variables such as Elevation, Tree cover, and Tree size, and its effects on HSV were already reflected in these variables.

**Table 2:** Major factors affecting habitat usage of *Rhinopithecus roxellana* during winter–spring and summer–autumn in Shennongjia District, China

Factors	Summer–autumn			Winter–spring		
	CR (%)	AUC	Range	CR (%)	AUC	Range
Elevation	25.9	0.75	1,750–3,100 m	42.1	0.86	2,100–2,600 m
Tree cover	25.1	0.77	0.5–0.9	0.2	0.77	0.7–0.9
Shrub height	12.4	0.77	1.4–3.0 m	14.7	0.80	2.0–3.0 m
Slope	11.5	0.70	<35°	4.5	0.68	<38°
Distance to water	7.9	0.59	<1,400 m	4.0	0.64	<1,750 m
Distance to road	5.4	0.64	>800 m	1.9	0.31	>200 m
Tree DBH	3.6	0.70	>9.0 cm	3.7	0.70	>11.0 cm
Tree height	1.9	0.76	>8.0 m	6.7	0.78	>11.0 m
Vegetation type	1.6	0.63	Mixed forest, conifer forest, broadleaves forest	0.1	0.76	Mixed forest, conifer forest
Shrub cover	0.1	0.69	0.35–0.7	13.0	0.77	0–0.35

Notes

Analysis of relative importance of major variables to habitat usage based on Maxent model.

AUC: area under the receiver operating characteristic curve; Contribution rate (CR%): percentage contribution of individual variable to habitat use; DBH: diameter at breast height; Range: the effective range of individual variables; Shrub cover: vertical projection of shrub canopy as percentage of land area; Tree cover: vertical projection of tree canopy as percentage of land area.

**Figure 3:**
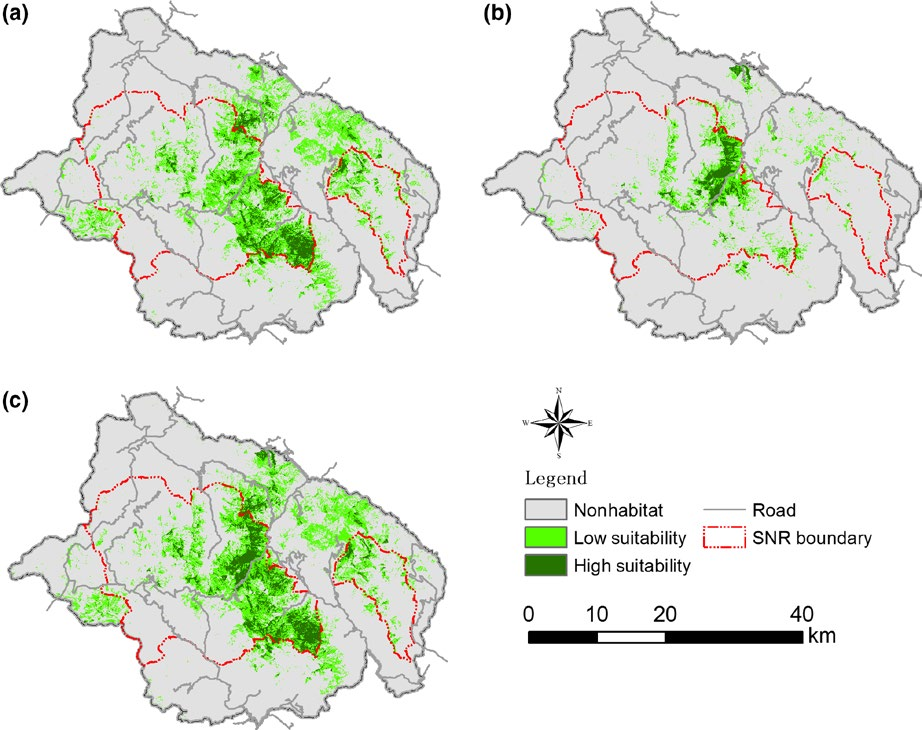
Spatial distribution of predicted potential habitat of *Rhinopithecus roxellana* in Shennongjia District, Hubei, China based on analysis of Maxent model. SNR: Shennongjia Nature Reserve. (a) summer–autumn; (b) winter–spring. (c) full year. Nonhabitat: HSI = 0–0.2, areas not suitable as habitat. Low suitability: HSI = 0.2–0.5, areas with low suitability as habitat. High suitability: HSI > 0.5, areas with high suitability as habitat

Spatial distribution of the predicted potential habitat varied between winter–spring and summer–autumn (Figure 3). Total areas with HSI > 0.2 were 126 km^2^ for winter–spring and 332 km^2^ for summer–autumn. Further, for winter–spring, over 80% of the areas with HSI > 0.5 were concentrated in areas north of Jianzhuling (Figure 3b). In comparison, areas with HSI > 0.5 were more widely distributed across the study area with high proportion located in areas south of Jianzhuling during summer–autumn (Figure 3a). The combined total areas with HSI > 0.2 for the two periods were 368 km^2^ (Figure 3c).

**Figure 4:**
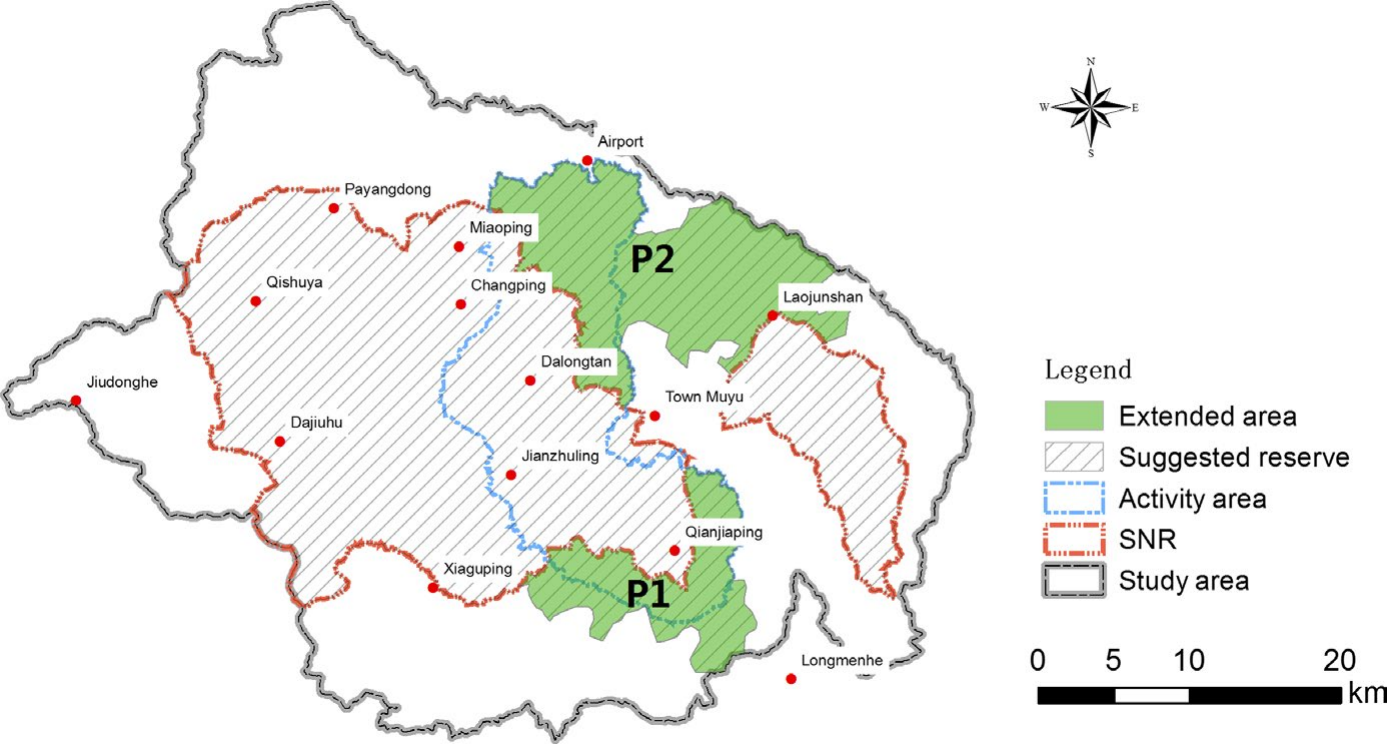
Recommended additions to the Shennongjia Nature Reserve (SNR), Shennongjia District, Hubei, China: P1 (84 km^2^) and P2 (186 km^2^). The activity area for *Rhinopithecus roxellana* is based on published records of Li et al. (2002), data of the SNR authority and data from the current study

Total habitat under protection of SNR was 202 and 91 km^2^ for the summer–autumn and winter–spring period, respectively (Figure 3a,b). When the two periods were combined, total protected habitat come to 224 km^2^ or 61% of the total predicted potential habitat area (Figure 3c). Three large patches of predicted potential habitat were outside the current SNR: an area between Laojunshan and Miaoping in the mid‐north, an area south of Qianjiaping in the mid‐south, and an area west of Dajiuhu to the far west of SNR (Figures 3c and 4).

Total habitat area of the golden snub‐nosed monkey was estimated to be 314 km^2^ including 130 km^2^ outside SNR (Figure 3c). Within this area, 189 km^2^ (60%) was areas with HSI = 0.2–0.5 and 74 km^2^ with HSI > 0.5, which accounted for 78% of the total predict habitat in this category in Shennongjia District. Two large patches of predicted potential habitat, one north and northwest of Laojunshan to the east of the study area and the other to the west of Changping, were outside the current habitat area (cp Figure 3c and 4).

To maximize the success of population conservation of *R. roxellana*, the reserve would need to be enlarged to encompass two additional areas: P1, south of Qianjiaping in the southern part of the Shennongjia District; and P2, between Laojunshan and Miaoping in the north (Figure 4). P1 would include all of the habitat area south and southeast of Qianjiaping (84 km^2)^ that is currently outside SNR. P2 would encompass all the northern habitat area between Laojunshan and east of Miaoping (186 km^2^) that is also currently outside SNR. Total area of an enlarged reserve would be 993 km^2^, an increase of 270 km^2^. The enlarged reserve would encompass 100% of the existing habitat area and 89% of the predicted potential habitat area (Figure 4).

## 4 DISCUSSION

### 4.1 Conservation effectiveness of SNR

Conservation effectiveness of reserves can be assessed using different approaches and different criteria. For some species, indices based on minimal viable population and minimal habitat requirements are used to assess effectiveness of given reserves or areas (Karczmarski, Huang, & Chan, 2017; Mathews, 2016). For the golden snub‐nosed monkey, there is no reported minimal viable population. The monkeys live in group of multiple males with each male partnering with one or several females of the group; other males may join the group at various time hence group size varies considerably (Pan et al., 2005). Such behavior of the monkeys has resulted in relatively low level of genetic polymorphism (Pan et al., 2005), thus the need to build up a population as large as possible to ensure the long‐term survival of the species. In our study, conservation effectiveness of SNR for this species was assessed with a niche‐based approach. Our analysis showed that SNR protects only 61% of the predicted potential habitat area (high value area) and 59% of the current habitat area of the golden snub‐nosed monkeys. While this result may compare favorably to those reported by Pressey and Tully (1994), Runte (1997), Holdgate (2014), and Pavez‐Fox and Estay (2016), further analysis revealed two shortcomings of SNR under its current management practices. First, as populations of the species tended to move to different areas between winter–spring and summer–autumn in response to availability of food and shelter, 41% of the current habitat areas was outside SNR and unprotected (Figure 3c). When outside SNR, the monkeys are in danger of being illegally hunted or being harmed/killed by agriculture, forestry, and other anthropogenic activities. Second, of the 368 km^2^ of predicted potential habitat areas, only 189 km^2^ were current area used by the monkeys (Figure 3c). In other words, 49% of the potential habitat areas are currently not used by the monkeys. The aggregation of the monkeys in relatively small areas may also result in overexploitation of local resources. We observed that the health of the *P. armandii* forest was severely affected by foraging and other activities of the monkeys in the Dalongtan area (unpublished observations). It is unknown whether crowding constrains population growth of these animals. Therefore, further study of the population ecology of the monkeys would be highly desirable.

With nearly half of available habitat outside the current activity area of the golden snub‐nosed monkey, there is likely to be habitat fragmentation and poor connectivity between the current activity areas and other patches of potential habitat. For instance, we found no activity of the monkeys in the Laojunshan area east of the township of Muyu despite the existence of large patches of suitable habitat (Figure 3c). Connectivity between the Laojunshan area and the current golden monkey populations was affected by the development of Muyu township in the middle and mid‐south sections and by agricultural and forestry activities in the northern section between these two areas. The area immediately north and northwest of Laojunshan is currently not part of SNR. The dominant vegetation in this area is evergreen and deciduous mixed forests and is potentially suitable as habitat for the monkeys (Figure 3c). Currently, the area is dotted with small patches of land being used for crop production, and as orchards and tea farms. The area is currently sparsely populated, and a high proportion of houses are dilapidated (unpublished observations) as most young people have left for towns and cities in search for better living and working conditions.

Habitat fragmentation can lead to population isolation which may result in high in‐breeding rates, low genetic diversity, and reduced fitness of the population. The reduction and fragmentation of habitat is responsible for population isolation and decline of many plant and wildlife species (Ashcroft, Gollan, & Batley, 2012; Fahrig, 2003; Krauss, Klein, Steffan‐Dewenter, & Tscharntke, 2004). High in‐breeding rate associated with isolated and small populations lowers the genetic diversity and reduces the fitness of the population and its ability to persist (Dixo, Metzger, Morgante, & Zamudio, 2009; Honnay & Jacquemyn, 2007; Valtonen et al., 2014). Habitat destruction is the number one cause of species endangerment, with 88% of all threatened and endangered species in the United States affected by habitat destruction (Noss, O'Connell, & Murphy, 1997). On a global scale, the loss of habitat has been identified as the single most important factor responsible for the extinctions of many wildlife species (30% of species extinctions have been attributed to habitat destruction: IUCN, 1992).

### 4.2 Key factors contributing to HSV

For both winter–spring and summer–autumn, our analysis suggests that elevation was the most important factor contributing to HSV of the golden snub‐nosed monkey. The usefulness of elevation as a predictor of potential distribution for mammals has been questioned by Hof, Jansson, and Nilsson (2012). Their analysis of published records showed that elevation is insignificant in predicting species distribution, more so in small regional areas. They argued that living organisms may not respond directly to altitudinal gradients but rather to other abiotic environmental factors regulated by elevation such as temperature and rainfall. Further, they pointed out that no obvious trends regarding taxa, spatial scale, resolution, and number of species studied, with regard to including or excluding elevation as a predictor variable. They showed that elevation is used as a predictor variable by just over half of the papers studied. Nonetheless, elevation appears to be an important variable contributing to seasonal movement of the golden snub‐nosed monkey in Shennongjia District. The predicted potential habitat was confined to elevation range of 1,750–3,100 m ASL for summer–autumn and 2,200–2,800 m ASL for winter–spring (Figure 3). These results were consistent with findings for golden snub‐nosed monkey in other areas. For instance, in Qingmuchuan Nature Reserve, habitat of golden the snub‐nosed monkey is located in elevation range of 1,400–3,400 m ASL and with seasonal variability (Li, Jiang, Li, & Grueter, 2010). The narrower elevation range in Shennongjia District is most likely to be due to human disturbance. Much of the Shennongjia District below 1,500 m was subject to intensive agricultural and forestry activities prior to the establishment of the reserve (Li, 2004; Li et al., 2007) and tourism more recently (Chang et al., 2012). During winter, the upper elevation range of the monkey was reduced to 2,800 m ASL in Shennongjia District. This downward movement of the upper elevation range was likely related to food availability and plant species composition. At elevations greater than 2,800 m ASL, vegetation in Shennongjia District is dominated by alpine meadow and fir forests and food for golden snub‐nosed monkey in these types of vegetation is scarce during the winter months. High elevation also placed extra pressure on the monkey during winter as temperatures are much lower (Luo et al., 2015).

The predicted potential habitat varies markedly between winter–spring and summer–autumn. About 18% of the study area (332 km^2^) was considered suitable as habitat for the monkey during summer–autumn (Figure 3). The area was reduced to 126 km^2^ during winter–spring. In other words, 62% of the areas that were considered suitable as habitat for the golden snub‐nosed monkey during summer–autumn became unsuitable during winter–spring. Similar change has been reported for the gray snub‐nosed monkey (*Rhinopithecus brelichi*) in Fanjing Mountain Biosphere Reserve (Wu, Wang, Fu, Zhao, & Yang, 2004). The gray snub‐nosed monkey has a defined habitat in the range of 1,500–1,700 m ASL in Fanjing Reserve during winter–spring. However, in summer, these monkeys were observed to move out of their winter–spring range into areas above 1,700 m ASL, where temperature is lower but food supplies are adequate. During winter, these monkeys moved downward to below 1,700 m ASL where food is more plentiful and temperature is warmer than at higher elevations.

Both Tree height and Tree DBH contributed to habitat selection of the golden snub‐nosed monkey. Areas with trees <10 m in height and <12 cm in DBH were generally avoided, whereas forests with trees of 10–20 m in height were preferred. These findings were consistent with earlier studies (Li, 2006; Li et al., 2002). This preference for larger trees is believed to be closely related to food availability and provision of shelter: Larger trees generally have more food (fruits and seeds and lichens) and offer better shelters from predators and adverse weather conditions than smaller ones (Li et al., 2002).

Shrub size (canopy cover and height) is important to habitat selection, especially during winter–spring (Table 2). In Shennongjia District, the monkeys fed primarily on lichens from barks of tree species, *Cerasus discadenia, P. armandii, Populus davidiana, Quercus glandulifera,* and *Salix wallichiana* from November to April (Li, 2006; Li et al., 2002). The influence of Shrub height and Shrub canopy cover on habitat selection is unlikely to be food related. The monkeys seek out areas of relatively low canopy cover (0–0.35) and avoid shrubs with high canopy cover, suggesting it maybe mobility related.

### 4.3 Proposed changes to improve conservation effectiveness of SNR

To maximize the success of population conservation of the golden snub‐nosed monkey, management should focus on the following three aspects. (a) Enlarging the current reserve to encompass the two additional areas: P1 and P2 (Figure 4). (b) Identifying and implementing measures to increase the connectivity between current activity area and other potential habitat areas to facilitate migration and recolonization of these areas. In the short‐term, construction of migration corridors and setting up road access restrictions will help the establishment of linkages with these potentially suitable habitat areas. In the long term, as vegetation of deforested areas regenerates, patches of fragmented habitat will join to form larger habitat areas, a potential reversal of habitat fragmentation. (c) Implementing a capture‐release program in an attempt to promptly recolonize suitable but currently unused habitat, particularly in the Laojunshan area. The obvious advantage of such a program is that it enables rapid occupation of new areas and the establishment of new populations of the monkeys.

The benefits of the proposed expansion are twofold. First, all of the activity area of the golden snub‐nosed monkey and 89% of the predicted potential habitat areas will be under the protection. Inclusion of all activity areas within the proposed reserve is a significant improvement on the current SNR. The monkeys are exposed to risk of illegal hunting and the influence of agricultural and forestry, and road traffic when outside the reserve. They are especially vulnerable during summer–autumn when they move down to lower elevations and enter areas not under the protection of SNR (Figure 2). Second, the inclusion of P2 to enable the connection of the Laojunshan area with the main part of the current SNR (Figure 4), making it possible for the monkeys to move to the Laojunshan area where there are large patches of potentially suitable habitat.

The proposed expansion represents a major trade‐off between socioeconomic interests and that of environmental protection. While a full feasibility study of the proposed reserve expansion is beyond the scope of the present study, it is envisaged that the expansion is possible both financially and socially. The recent approval of the Central Chinese Government to establish Shennongjia National Park from Shennongjia Nature Reserve is a significant development (http://www.ndrc.gov.cn/dffgwdt/201605/t20160530_805595.html) for the current SNR and its possible expansion. The new Shennongjia National Park will be administered directly under the Central and Provincial Governments with higher level of financial input and technical support. Historically, Shennongjia District has been sparsely populated at 23 people/km^2^ compared to 230–260 people/km^2^ of neighboring counties (Gong, Chen, & Zhang, 2015). As urbanization is accelerating, the number of people engaged directly in agricultural and forestry activities has declined at the rate of about 1000/year (Shennongjia Information Office 2015). By 2016, the number of people engaged in farming was 19,942, or about 6 people/km^2^. Many of the small family farms have become uneconomical and are abandoned. With financial support and policy direction from the National Government and the reduced number of people to relocate, the funding requirements of the proposed expansion can be easily accommodated. The proposed expansion is also more socially acceptable than it was 30 years ago. As living standard improved, people become more conscious of the importance of nature conservation and willing to accept incentives offered by governments (see Ma & Hu, 2010), so we anticipate the proposed expansion will be embraced willingly by the locals.

Furthermore, the proposed expansion is ecologically sound. Much of the proposed expansion area is mountainous with 87% of P1 and 81% of P2 (totaling 223 km^2^) made up of areas with slopes >15° (Figure 4). Under the Grain to Green Program (http://www.forestry.gov.cn/main/3031/content-860180.html), farming activities are prohibited in these areas and all of which are covered by forests. Of the remaining 47 km^2^, a large proportion is currently forest. In total, over 90% of the proposed expansion is forest. Thus, the proposed expansion will have enhanced the ecological integrity for the areas.

The ability to identify potential habitat areas for target species is fundamental for nature reserve design and selection (Le Saout et al., 2013; Morrison et al., 2012; Pressey et al., 1993). Using the niche‐based approach, we were able to integrate habitat usage data of the target species with that of remote sensing to identify areas potentially suitable as habitat for the species. This information can be used not only for improving conservation effectiveness of existing reserves but also for effective planning and designing of new reserves.

## 5 CONCLUSIONS

Many reserves, such as SNR, were established on limited information of the target species (or habitats) and were constrained by social and economic conditions at the time. Hence, areas that have the greatest conservation values may not have been included. Equal importantly, as population of target species changes in response to conservation management, their changing habitat requirements render existing reserves ineffective. Hence, there is a continuous need to review existing reserves in response to this change by the species under conservation. Our analysis illustrates that SNR currently only protect 61% of the available high value area for the golden snub‐nosed monkeys and only half of the potential habitat were used by the monkeys. Furthermore, 41% of the habitat area of the monkeys were outside the reserve, exposing the animals under risk of illegal hunting and influence of various anthropogenic activities. Enlargement of SNR by 270 km^2^ would allow the inclusion of 100% current habitat areas and 89% of the potentially suitable habitat area in the region, and thus greatly improve the conservation effectiveness of SNR for the golden snub‐nosed monkey. Using new information on the target species (or habitats) in the context of changed social and economic conditions, our study demonstrates how the adequacy of an existing reserve can be reassessed and inadequacies addressed to improve its effectiveness of conservation. Of equal importance, our study has also shown how niche‐based models, such as Maxent, can be used to identify potential habitat for a target species and can facilitate nature reserve design and selection for conservation of that species.

## CONFLICT OF INTEREST

The authors declare no conflict of interest.

## AUTHOR CONTRIBUTIONS

PW and MT designed the study, YW and JY conducted the fieldwork, and WH and ZY performed the analyses. All six authors wrote the manuscript.

## DATA ACCESSIBILITY

Field sampling data to be archived at Dryad upon acceptance of the manuscript.
